# Glucocerebrosidase Defects as a Major Risk Factor for Parkinson’s Disease

**DOI:** 10.3389/fnagi.2020.00097

**Published:** 2020-04-21

**Authors:** Micol Avenali, Fabio Blandini, Silvia Cerri

**Affiliations:** ^1^Neurorehabilitation Unit, IRCCS Mondino Foundation, Pavia, Italy; ^2^Department of Brain and Behavioural Sciences, University of Pavia, Pavia, Italy; ^3^Laboratory of Cellular and Molecular Neurobiology, IRCCS Mondino Foundation, Pavia, Italy

**Keywords:** Parkinson’s disease, glucocerebrosidase, alpha-synuclein, GBA1, risk factor, synucleinopathy

## Abstract

Heterozygous mutations of the GBA1 gene, encoding for lysosomal enzyme glucocerebrosidase (GCase), occur in a considerable percentage of all patients with sporadic Parkinson’s disease (PD), varying between 8% and 12% across the world. Genome wide association studies have confirmed the strong correlation between PD and GBA1 mutations, pointing to this element as a major risk factor for PD, possibly the most important one after age. The pathobiological mechanisms underlying the link between a defective function of GCase and the development of PD are still unknown and are currently the focus of intense investigation in the community of pre-clinical and clinical researchers in the PD field. A major controversy regards the fact that, despite the unequivocal correlation between the presence of GBA1 mutations and the risk of developing PD, only a minority of asymptomatic carriers with GBA1 mutations convert to PD in their lifetime. GBA1 mutations reduce the enzymatic function of GCase, impairing lysosomal efficiency and the cellular ability to dispose of pathological alpha-synuclein. Changes in the cellular lipidic content resulting from the accumulation of glycosphingolipids, triggered by lysosomal dysfunction, may contribute to the pathological modification of alpha-synuclein, due to its ability to interact with cell membrane lipids. Mutant GCase can impair mitochondrial function and cause endoplasmic reticulum stress, thereby impacting on cellular energy production and proteostasis. Importantly, reduced GCase activity is associated with clear activation of microglia, a major mediator of neuroinflammatory response within the brain parenchyma, which points to neuroinflammation as a major consequence of GCase dysfunction. In this present review article, we summarize the current knowledge on the role of GBA1 mutations in PD development and their phenotypic correlations. We also discuss the potential role of the GCase pathway in the search for PD biomarkers that may enable the development of disease modifying therapies. Answering these questions will aid clinicians in offering more appropriate counseling to the patients and their caregivers and provide future directions for PD preclinical research.

## Introduction

Parkinson’s disease (PD) is the second most common neurodegenerative disorder after Alzheimer’s disease, with a 0.3% prevalence in the general population, increasing to 1% in people older than 60 years and to 3% in the over 80s (Lee and Gilbert, 2016).

PD is characterized by a wide spectrum of motor and non-motor symptoms (Lee and Gilbert, 2016). According to clinical and imaging findings, the neurodegenerative process of PD may begin 7–10 years before the appearance of the classic motor symptoms bradykinesia, rigidity, and tremor (Hawkes, 2008; Schapira and Tolosa, 2010). Non-motor symptoms, a crucial component of the PD clinical picture, include olfactory loss, rapid eye movement sleep behavior disorders (RBD), constipation, dysautonomia, and depression, and can precede PD motor symptoms by many years (Schapira and Tolosa, 2010; Schapira et al., 2017; Heinzel et al., 2019).

The majority of PD cases are idiopathic, with a 10–15% fraction being associated with gene mutations (Sidransky et al., 2009; Westbroek et al., 2011; Schapira, 2015). In the context of PD genetics, the attention of many groups have converged, in recent years, onto the GBA1 gene, which encodes glucocerebrosidase (GCase), a lysosomal enzyme that breaks down glucocerebroside into glucose and ceramide (Beutler et al., 2005; Grabowski, 2008).

Homozygous mutations in the GBA1 gene cause Gaucher’s disease (GD), which may present with either neurological involvement (neuronopathic) or not (non-neuronopathic). Interest in GBA1, as a causative factor for PD, was initially sparkled by the observation, in the 1990s, that among GD patients and GBA1 mutation carriers, a higher proportion developed parkinsonian symptoms (Neudorfer et al., 1996; Machaczka et al., 1999). Moreover, the association between GBA1 mutation and PD development was confirmed by a large multicenter study demonstrating a significantly higher prevalence of GBA1 mutations among the PD population (Sidransky et al., 2009). Although the proportion of PD patients with GBA1 mutations varies by ethnicity and sequencing methods used, recent studies suggested that the heterozygous status confers a cumulative risk of developing PD of 5% at age 60, rising to 15–30% at age 80 (Schapira, 2015; Balestrino and Schapira, 2018). The GBA1 mutation prevalence is estimated between 2.35% and 9.4% in the PD population, climbing to 31.3% in the PD Ashkenazi Jewish (AJ) population (Schapira, 2015) indicating these mutations as the most common genetic risk factor for PD.

GD patients and asymptomatic heterozygous GBA1 mutation carriers share the same risk of developing PD (Alcalay et al., 2012), but not all GBA1 mutant carriers will develop the disease. Interestingly, PD risk seems to be affected by GBA1 mutations with varying degrees of severity (Sidransky and Lopez, 2012; Migdalska-Richards and Schapira, 2016).

GBA1 mutations reduce the enzymatic function of GCase, which may favor toxic accumulation of alpha-synuclein fibrils in the typical intra-neuronal inclusions (Lewy bodies) found throughout the central nervous system of PD patients (Balestrino and Schapira, 2018). Alternatively, or in addition to, changes in the cellular lipidic content, resulting from the accumulation of glycosphingolipids associated with lysosomal dysfunction, may contribute to the pathological modification of alpha-synuclein. Various classes of lipids can strongly interact with alpha-synuclein, causing modifications of its native structure, which can favor the formation of fibrils and subsequent aggregation of the protein (Morabito et al., 2017; Fecchio et al., 2018).

GCase defects may also contribute to neuroinflammation, a phenomenon that is increasingly implicated in PD pathogenesis, as suggested by various studies demonstrating that a reduction in GCase activity is associated with clear activation of microglia in the brain parenchyma (Ginns et al., 2014; Rocha et al., 2015c; Soria et al., 2017; Mus et al., 2019).

Moreover, GCase deficiency has been observed in idiopathic PD (iPD) patients without GBA1 mutation, suggesting a central, more widespread involvement of this enzyme in PD pathogenesis (Chiasserini et al., 2015; Parnetti et al., 2017).

From a clinical point of view, GBA1-associated PD (GBA-PD) is very similar to iPD, except for an earlier onset and higher prevalence of cognitive deterioration and non-motor features, manifesting before the development of the PD motor features (Siebert et al., 2014). The risk of cognitive impairment in GBA-PD patients is 2.4-fold higher than in non-carriers (Nalls et al., 2013). GBA1 heterozygous carriers have an increased chance of developing either PD or dementia with Lewy bodies at an earlier age of onset, a higher risk for progression to dementia, visual hallucinations, autonomic dysfunction and faster progression of motor symptoms than non-carriers, and an overall decreased survival rate (Winder-Rhodes et al., 2013; Cilia et al., 2016).

GBA1 mutations causing GD have been categorized as “severe” (L444P, for example) or “mild” (N370S, for example), with a residual GCase activity of 13–24% and 32–38%, respectively (Beutler et al., 2005). N370S and L444P mutations are the two most relevant GBA1 mutations in the PD population although, recently, the E236K mutation, which is absent in the GD population, has been identified as the most prevalent PD-associated GBA1 mutation (Duran et al., 2013). Mutation severity can influence PD phenotype profoundly. A meta-analysis revealed that the risk for dementia in PD patients carrying “severe” mutations is 2- to 3-fold higher than in those carrying “mild” mutations (Cilia et al., 2016). A more recent study extended this notion demonstrating that psychiatric symptoms, cognitive impairment, and olfactory deficiency are more pronounced in PD patients carrying severe GBA1 mutations (Thaler et al., 2018a).

In this review article, we summarize the current knowledge on the multiple roles that GBA1 mutations, and resulting GCase/lysosomal impairment, may play in the pathogenesis of PD and their phenotypic correlations. We also discuss the potential role of the GCase pathway in the search for PD biomarkers and molecular targets that may help the development of disease modifying therapies.

## Neuropathological Potential Mechanisms of GBA-PD

The mechanism by which GBA1 mutations are linked to PD is still poorly understood. However, as in iPD, several mechanisms may be involved in both the development and progression of GBA-PD, such as alpha-synuclein accumulation, neuroinflammation, mitochondrial deficiency, autophagic dysfunction, and oxidative stress (Schapira and Tolosa, 2010; Blandini et al., 2019).

### Glucocerebrosidase and Alpha-Synuclein

The relationship between alpha-synuclein accumulation and GCase is still a matter of study. Experimental findings and clinical observations led to three main hypotheses that may explain this relationship. These hypotheses may not be mutually exclusive and multiple mechanisms might be in place.

The *gain-of-function hypothesis* suggests that misfolded GCase directly interacts with alpha-synuclein, leading to an increase of alpha-synuclein accumulation and aggregation (Sidransky and Lopez, 2012). Indeed, GCase was observed in abnormal protein aggregates, such as Lewy bodies and neurites, from GBA-PD brains, confirming a co-localization of mutant GCase and alpha-synuclein *in vivo* (Goker-Alpan et al., 2008). Cullen et al. (2011) reported increased alpha-synuclein levels in GBA mutant neural cells. This finding was confirmed by Xu et al. (2011) in the forebrain and cerebellum of mice carrying the homozygous V394L GBA1 mutation. However, this hypothesis is at odds with the general observation that congenital diseases involving enzymes almost exclusively cause a loss of function and that null GBA1 mutations are associated with PD risk (Neumann et al., 2009; Gan-Or et al., 2015; Franco et al., 2018).

According to the *loss-of-function hypothesis*, GBA1 mutations may affect GCase protein structure, leading to decreased enzymatic activity *via* multiple mechanisms (e.g., the failure of the GCase protein to exit the endoplasmic reticulum or to link with its transporter). The reduction in GCase activity causes an accumulation of its substrates with subsequent perturbation of lipid homeostasis. This affects trafficking, processing, and clearance of alpha-synuclein, resulting in accumulation and aggregation of the protein (Mazzulli et al., 2011; Westbroek et al., 2011; Sidransky and Lopez, 2012; Suzuki et al., 2007; PMID: 26362253). In line with this, Rocha et al. (2015a) observed that increased levels of glucosylsphingosine are associated with reduced GCase activity in the substantia nigra and hippocampus of iPD patients. Daily systemic treatment with selective GCase inhibitor conduritol-β-epoxide (CBE) in mice promoted the accumulation of lipid substrates and insoluble α-synuclein aggregates in the substantia nigra (Rocha et al., 2015a). Huebecker et al. (2019) recently reported a significant increase in glucosylceramide levels in the substantia nigra of iPD patients in association with deficiencies in multiple lysosomal hydrolases, including GCase, α-galactosidase, and β-hexosaminidase. These findings partially contradict previous studies that did not report GCase substrate accumulation in the brain of PD patients with heterozygote GBA1 mutations or iPD patients with reduced GCase activity (Murphy et al., 2014; Gegg et al., 2015). These discrepancies are not easy to reconcile, but might be due, at least in part, to differences in the brain regions examined and/or to the different methodologies used to measure lipid substrates. Still at odds with the *loss-of-function hypothesis*, pharmacological inhibition of GCase activity by CBE in cells overexpressing alpha-synuclein and primary neurons did not alter alpha-synuclein levels or solubility (Zurbruegg et al., 2019; Henderson et al., 2020). On the contrary, siRNA knockdown of the GBA1 gene significantly increased alpha-synuclein levels, suggesting that alpha-synuclein accumulation is unlikely to be solely due to decreased GCase activity (Zurbruegg et al., 2019).

A *third hypothesis* proposes a bidirectional loop in which GCase deficiency facilitates alpha-synuclein oligomer formation that, in turn, impairs GCase activity, ultimately promoting further formation of alpha-synuclein oligomers (Mazzulli et al., 2011). Accordingly, overexpression of exogenous alpha-synuclein in *in vitro* models causes reduction of GCase activity and protein levels (Gegg et al., 2012). In addition, Wong and Krainc (2016) have demonstrated that loss of GCase activity in human midbrain neuron cultures promotes alpha-synuclein accumulation and toxicity, which in turn disrupts trafficking to lysosomes of GCase and additional lysosomal hydrolases, further contributing to GCase deficits and—more in general—to lysosomal dysfunction. This hypothesis is not completely supported by *in vivo* studies, since no changes in GCase protein levels and activity were observed in the brain of different mouse models overexpressing human wild-type or A53T (M83KO) alpha-synuclein (Richter et al., 2014; Henderson et al., 2020). In addition, it should be noted that build-up of alpha-synuclein has been documented in many lysosomal disorders (LSD; Saito et al., 2004; Suzuki et al., 2007; Nelson et al., 2014; Smith et al., 2014). In Krabbe disease, a demyelinating LSD caused by deficiency in galactosylceramidase, the accumulation of substrate psychosine would promote the disruption of lipid raft architecture, which in turn may affect alpha-synuclein localization to synapses and increase its aggregation in the neuronal cytoplasm (Smith et al., 2014). Phosphorylated alpha-synuclein accumulation was found in the brain of patients with Niemann-Pick type C1 disease as a consequence of defects in intracellular cholesterol trafficking (Saito et al., 2004). Together, these findings indicate that, as in LSD, lipid accumulation could trigger a cascade of events driving PD pathology.

Interestingly, a recent study highlighted the importance of the lysosomal protein cathepsin D in mediating the increase of monomeric alpha-synuclein levels associated with GBA1 mutations, indicating a new player in the relationship between GCase and alpha-synuclein (Yang et al., 2020).

Despite the large body of evidence in favor of the link between GCase deficiency and alpha-synuclein accumulation, the question of why only a small proportion of individuals with GBA1 mutations develops PD remains unanswered, suggesting that other factors must be playing a role.

### Glucocerebrosidase and Mitochondria Dysfunction

Mitochondria dysfunctions have been classically involved in the pathogenesis of iPD (Schapira et al., 1989, 1990; Schapira, 2008; Mounsey and Teismann, 2011; Schapira and Gegg, 2011) and may also be a feature of GBA PD pathobiology. Cleeter et al. (2013) demonstrated that GCase loss-of-function triggers oxidative stress and mitochondrial dysfunction in human dopaminergic cell lines, together with an accumulation of alpha-synuclein. Similar findings were observed in *in vivo* models of GCase deficiency. Osellame et al. (2013) found an accumulation of dysfunctional mitochondria with subsequent reduction in respiratory chain complex activities and oxygen consumption in a mouse model carrying homozygous knock-out of the GBA1 gene.

Similarly, a reduction of oxygen consumption and mitochondrial adenosine triphosphate production was demonstrated in neuronopathic GD mouse models generated from backcrossing of the homozygous point-mutated GBA1 mice with hypomorphic prosaposin mice (Xu et al., 2014).

Recently, Li et al. (2019) investigated the link between heterozygous GBA1 mutation and mitochondrial dysfunction showing that in GBA1 L444P/WT knock-in mice, the GBA1 mutation triggers mitochondrial dysfunction by blocking autophagy and mitochondrial priming. Moreover, Schöndorf et al. (2018) demonstrated that neurons differentiated from GBA-PD patients induced pluripotent stem cells showed alterations in morphology, energy metabolism, and mitochondrial function.

### Glucocerebrosidase and Endoplasmatic Reticulum Stress

Endoplasmic reticulum-associated degradation (ERAD) promotes balanced cell proteostasis, degrading altered proteins through ubiquitination and proteosomal degradation (Migdalska-Richards and Schapira, 2016). ERAD impairment leads to the accumulation of misfolded proteins, promoting ER stress and apoptosis.

Emerging evidence shows that ER stress has a pivotal role in the pathobiology of PD (Mercado et al., 2013; Sardi et al., 2015), but few studies have analyzed, so far, the link between ER stress and the pathogenesis of GBA-PD. Mutant misfolded GCase triggers ER stress and evokes proteasomal degradation (Ron et al., 2010; Maor et al., 2013). Indeed, a study on human GD fibroblasts showed misfolded GCase retained in the ER (Bendikov-Bar et al., 2011). Moreover, the degree of GCase retention in ER and proteasomal degradation seems to be correlated with GD severity (Ron and Horowitz, 2005). Gegg et al. (2012) found that ERAD was increased in the GBA-PD brains. Furthermore, a study using induced pluripotent stem cells from GBA1 carriers confirmed increased ER stress and ERAD (Fernandes et al., 2016).

Interestingly, blocking GCase activity with CBE also stimulated ER stress in neuroblastoma cells, suggesting that GCase activity reduction, also in the absence of a GBA1 mutation, may play a role in this context (Gegg and Schapira, 2018).

### Glucocerebrosidase and Neuroinflammation

Neuroinflammation—mostly expressed as microglia activation—is clearly implicated in PD progression and accompanies dopaminergic cell death since the very early phase of the disease (Fuzzati-Armentero et al., 2019). GCase defects may substantially contribute to this phenomenon. Marked increases in the level of inflammatory mediators have been reported in the brain of a neuronopathic GD mouse model (Vitner et al., 2012). Subsequent studies have further detailed how a reduction in GCase activity is associated with clear glial activation. Ginns et al. (2014) reported astrocytosis and microglia activation in the nigrostriatal tract of GBA1-mutant mice, as well as in mice treated sub-chronically with GCase inhibitor CBE, which also induced alpha-synuclein accumulation. These results were confirmed by the studies from Rocha et al. (2015b) and Mus et al. (2019), both showing that a CBE-induced drop in GCase enzymatic activity activates microglia throughout the brain. Interestingly, using a DAT-GBA1-KO mouse displaying selective homozygous GBA1 disruption in midbrain dopamine neurons, Soria et al. (2017) demonstrated strong microglial activation in the substantia nigra of these mice, without any evidence of dopaminergic cell loss, alpha-synuclein accumulation, or motor abnormalities.

## Pathogenic Mutations of GBA1 Associated PD

More than 300 mutations in the GBA1 gene, such as insertions, deletions, and point mutations, have been discovered so far (O’Regan et al., 2017; Zhang et al., 2018a). The N370S (c.1226A > G) and the L444P (c.1448T > C) mutations are the most common mutations worldwide (Zhang et al., 2018b). A recent meta-analysis described other GBA1 variants, such as R120W, IVS2 + 1G > A, H255Q, D409H, RecNciI, E326K, and T369M related to PD risk. Ethnic heterogeneity of GBA1 mutations in PD was also reported. R496H and 84insGG increase PD risks exclusively in AJ populations, while L444P, E326K, T369M, R120W, IVS2 + 1G > A, H255Q, D409H, and RecNciI were found more frequently in non-AJ subjects. N370S is correlated to an increased PD risk in all populations (Zhang et al., 2018b).

## Prodromal Features in GBA1 Mutation Carriers

A substantial body of evidence suggests the existence of a prodromal phase of iPD, of variable length, preceding the onset of the classical signs of the disease (Schapira and Tolosa, 2010; Schapira et al., 2017; Heinzel et al., 2019). The existence of such a premotor period has been proposed based on imaging, pathology, clinical, and epidemiological studies, suggesting that the nigrostriatal lesion might evolve in 7–10 years before becoming clinically manifest (Hawkes, 2008; Schapira and Tolosa, 2010). Although stiffness, tremors, or imbalance may be present as prodromal features (de Lau et al., 2006), the vast majority of early symptoms and signs in PD are of the non-motor type, including hyposmia, REM behavior disorder (RBD), constipation, depression, and color discrimination (Hawkes, 2008; Postuma and Berg, 2016; Schapira et al., 2017).

A prodromal phase was also described in both GD and heterozygous GBA1 carriers without a clinical diagnosis of PD (McNeill et al., 2012a,b). Cognitive and olfactory functions were significantly impaired and motor testing was abnormal in GD patients and GBA1 mutation carriers without PD, compared to healthy subjects. No differences were found in terms of sleep abnormalities or autonomic function (Beavan et al., 2015; Avenali et al., 2019).

In a web-based evaluation of prodromal markers designed to estimate PD risk, Noyce et al. (2014, 2015) observed that the odds of having a GBA1 variant was 9.5 times higher in the “high risk group” compared to other groups.

## Clinical Features in GBA-PD

**Figure 1:**
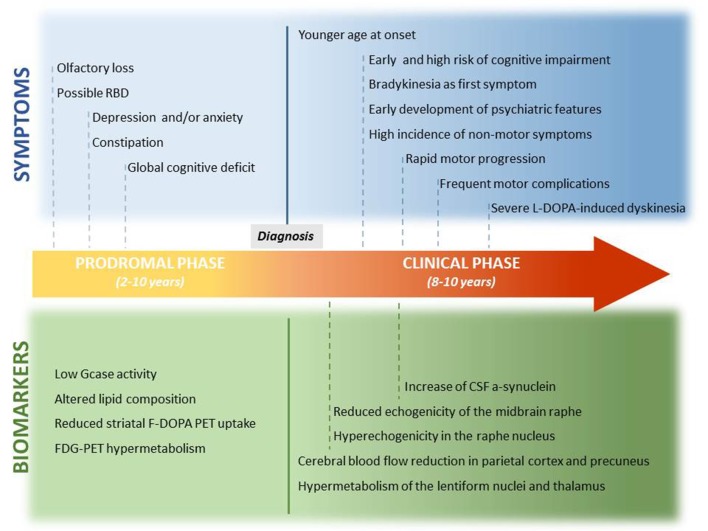
Prodromal and clinical features of GBA-PD and related biomarkers. This is a graphic representation of the potential timeline by which the preclinical symptoms and motor and non-motor features of GBA-PD may manifest. Potential biomarkers are also reported, and they are correlated with the onset of clinical features.

Compared to iPD, GBA-PD patients are characterized by a slightly younger age at onset, higher incidence of neuropsychiatric features (such as hallucination, depression, and anxiety), greater incidence of sleep disturbances, and earlier development of cognitive deficits (Neumann et al., 2009; Sidransky et al., 2009; Brockmann et al., 2011; McNeill et al., 2012b; Winder-Rhodes et al., 2013). In general, the disease course tends to be more aggressive in GBA-PD patients (Winder-Rhodes et al., 2013; Zokaei et al., 2014; Brockmann et al., 2015b; Cilia et al., 2016; Jesús et al., 2016; Figure 1).

### Motor Features

GBA-PD patients exhibit an asymmetric onset with the classic triad of motor symptoms, bradykinesia, rigidity, and tremors (Goker-Alpan et al., 2008), although bradykinesia seems to be more common, as an initial symptom, than in iPD (Ziegler et al., 2007; Gan-Or et al., 2010; Lesage et al., 2011).

Longitudinal cohort studies using the Unified Parkinson’s Disease Rating Scale, motor subscale, and Hoehn and Yahr staging showed a more rapid motor progression in GBA-PD than iPD patients (Winder-Rhodes et al., 2013; Brockmann et al., 2015b). One study in a large European series (Lesage et al., 2011) reported that L-Dopa induced dyskinesias were more severe in GBA-PD patients, but no difference was observed in another study (Zhang et al., 2015).

Motor complications, such as swallowing disorders, dysarthria, and freezing of gait, are observed more frequently in GBA-PD patients (Jesús et al., 2016). The risk of developing motor fluctuations and dyskinesia seems to be higher in GBA-PD subjects carrying a severe mutation (Cilia et al., 2016; Jesús et al., 2016; Lythe et al., 2017).

### Non-motor Features

Non-motor symptoms are the earliest presenting symptoms in GBA-PD, as well as in iPD, but some of them may be more common in GBA-PD patients.

Compared with iPD, GBA-PD patients show a higher risk for cognitive deterioration (Gan-Or et al., 2010). Several studies investigated the difference in cognitive performance between iPD and GBA-PD patients (Goker-Alpan et al., 2008; Alcalay et al., 2012; McNeill et al., 2012b; Nalls et al., 2013; Malec-Litwinowicz et al., 2014; Cilia et al., 2016). In a meta-analysis conducted by Ziegler et al. (2007), the risk of cognitive impairment was three times higher for patients with GBA1 mutations. Cognitive impairment seems to affect memory and visuo-spatial domains (Alcalay et al., 2012), working memory, executive and visuospatial abilities (Mata et al., 2016), and visual short-term memory (Zokaei et al., 2014) in particular. GBA-PD patients may undergo cognitive decline sooner than iPD patients (Winder-Rhodes et al., 2013; Brockmann et al., 2015b; Oeda et al., 2015; Liu et al., 2016), although this evidence was not reported by all studies (Aharon-Peretz et al., 2005; Clark et al., 2007; Nichols et al., 2009).

GBA-PD shows increased frequency of psychiatric symptoms at an earlier stage (Oeda et al., 2015; Cilia et al., 2016), as well as a higher risk for delirium and hallucinations when compared with iPD (Aharon-Peretz et al., 2005; Wang et al., 2014), without differences linked to mutation severity (Barrett et al., 2014).

Olfactory functions are affected in GBA1 mutation carriers, as they are in early iPD patients (McNeill et al., 2012b).

A substantial impact of depression was initially reported in GBA-PD patients (Machaczka et al., 1999), but was not confirmed by subsequent studies (Winder-Rhodes et al., 2013; Zhang et al., 2015). Analogously, conflicting results have been reported for anxiety and apathy, whose frequency in GBA-PD patients was found to be increased (Brockmann et al., 2011; Wang et al., 2014) compared to iPD patients.

Dysautonomic symptoms such as bowel alteration, sexual impairment, orthostatic symptoms, and urinary dysfunction are other non-motor symptoms frequently reported in GBA-PD individuals (Brockmann et al., 2011; Wang et al., 2014).

As for pain, contradictory findings have been reported, without a clear indication that pain may afflict GBA-PD more than iPD patients (Gan-Or et al., 2010; Kresojević et al., 2015).

### Genotype-Phenotype Correlation in GBA-PD Subjects

GBA1 mutations may have differential effects on PD risks, depending on the specific variant, as well as on the clinical profile and disease progression rate. For mild GBA1 mutation carriers, the odds ratios for developing PD ranged between 2.84 and 4.94, while for severe GBA1 mutation carriers the odds ratios were between 9.92 and 21.29 (Gan-Or et al., 2015). The risk for dementia in GBA-PD subjects bearing severe mutations (L444P, splicing mutation IVS10 + 1G > T) is 5.6 times greater than in iPD patients and 2.9 times greater than in patients carrying mild GBA1 mutations (N370S; Cilia et al., 2016). The risk of hallucination development is not affected by the severity of GBA1 mutations (Barrett et al., 2014).

In a more recent study, motor and some non-motor features (depression, RBD, and olfactory loss) were significantly worse in GBA-PD patients with severe mutations than in those bearing mild mutations (Thaler et al., 2018a).

E326K is the most prevalent PD-associated GBA1 mutation and has not been described in GD (Duran et al., 2013). PD patients bearing E236K mutations show a faster progression of motor symptoms, with gait disorders and postural instability. They also have a higher risk of cognitive decline, but a lesser risk for motor complications (Winder-Rhodes et al., 2013; Davis et al., 2016; Mata et al., 2016).

Data on genotype-phenotype correlations between other GBA1 polymorphisms, clinical features, and risk rate of progression are lacking. Further research is therefore mandatory, in order to better clarify the role of GBA1 variants in specific clinical manifestations and disease progression.

## Biomarkers

No reliable biomarker that may inform on the ongoing neuronal loss, before the onset of PD clinical features, is yet available. The knowledge that has been accumulating on GBA1 defects in PD may help us fill this gap (Figure 1).

### Neuroimaging

Positron emission tomography (PET) employing several different radioisotopes [fluorodeoxyglucose (FDG), F-Dopa, and C-CFT, 2β-carbomethoxy-3β-(4-fluorophenyl-tropane)] has been used to assess brain metabolism in GBA-PD. Kono et al. (2010) first described a significant FDG decrease in the supplementary motor area of the frontal cortex in GBA1 mutants (one GD-PD patient, two GBA-PD patients, and three GBA1 heterozygous carriers). GBA-PD patients also showed hypometabolism in the parieto-occipital cortices, while GBA heterozygous carriers without PD showed normal metabolism in the putamen and increased metabolism in the caudate nucleus (Kono et al., 2010). Using a different tracer (^11^C-CFT, binding to the dopamine transporter) the same group also demonstrated a significant presynaptic dysfunction in GBA-PD, with a severe reduction of striatal uptake in both putamen and caudate nucleus, whereas asymptomatic GBA carriers had increased caudate uptake with normal putamen uptake (Kono et al., 2010).

Fluorodopa PET or single-photon emission computerized tomography (SPECT) with dopamine-sensitive ligands in GD-PD patients showed asymmetric striatal dopaminergic neuronal loss with the highest reduction in the caudate and posterior putamen nuclei; this reduction was also observed in iPD brains, although less represented (Goker-Alpan et al., 2012). By analyzing the resting regional cerebral blood flow, the same authors reported bilateral hypoperfusion in the lateral parieto-occipital association cortex and precuneus in GD-PD, but not in iPD patients. They also demonstrated that in GD subjects without PD-like manifestations, the F-Dopa uptake was decreased in the striatum, with a marked neuronal loss in the tail of the putamen, whereas this reduction was not found in GBA1 heterozygous asymptomatic carriers (Goker-Alpan et al., 2012). More recently, a large cohort study conducted within the Parkinson’s Progression Markers Initiative of the Michael J. Fox Foundation reported increased DAT striatal binding ratios in GBA asymptomatic carriers, compared with healthy controls (Simuni et al., 2020).

A magnetic resonance spectroscopy study conducted by Brockmann et al. (2012) showed significant reduction of N-acetylaspartate and choline levels in the putamen and in the midbrain of GBA-PD patients, compared to healthy subjects, along with increased levels of glycerophosphoethalonamine, pointing to a reduction in neuronal integrity and altered membrane phospholipid metabolism.

As for MRI structural data, Agosta et al. (2013) reported a diffused pattern of white matter alterations involving the parahippocampal tract and the parietal and occipital lobes, including interhemispheric and frontal cortical connections, in GBA-PD patients compared to iPD patients. On the other hand, when cortical thickness and subcortical volumes were more recently analyzed, no differences between GBA-PD and iPD patients were observed (Thaler et al., 2018b).

Transcranial sonography is able to detect substantia nigra hyperechogenicity in 90% of iPD patients and has been proposed as a potential screening tool for the early detection of PD (Beavan and Schapira, 2013). The few studies conducted on GBA1 positive individuals (Saunders-Pullman et al., 2010; Brockmann et al., 2011; Barrett et al., 2013) reported a greater substantia nigra echogenicity when compared to healthy controls, but without any differences with iPD. More recently, Arkadir et al. (2019) reported an enlarged area of hyperechogenicity also in the substantia nigra of asymptomatic GBA1 carriers and GD patients without PD, with respect to healthy controls.

Finally, myocardial ^123^I-metaiodobenzylguanidine scintigraphy showed a marked reduction of tracer accumulation in PD patients carrying GBA1 mutation compared to iPD patients (Li et al., 2014), suggesting that neurodegeneration of myocardial nerve fibers had begun before clinical presentation and/or that disease progression is more rapid in this population. Reduced myocardial uptake of the tracer was also demonstrated by Oeda et al. (2015) in GBA-PD patients, but in this case without differences with respect to iPD.

Although several imaging biomarkers have been proposed to discriminate PD subjects with and without GBA1 mutations, no conclusive evidence has yet been produced. Further imaging studies on wider populations will be needed to better clarify the neuroimaging correlates of GBA-PD.

### Biochemical Markers

Several studies investigated the possible value of GCase enzymatic activity as a potential biomarker for PD. Decreased GCase activity has been observed in fibroblasts and in the cerebrospinal fluid (CSF) from GD, GBA-PD, and also iPD patients (Parnetti et al., 2009, 2014; Gegg et al., 2012; Ambrosi et al., 2015). Moreover, Parnetti et al. (2014) observed that PD patients at an early stage of the disease showed lower GCase activity levels than patients at later stages, suggesting that GCase could have a potential role as a marker of early PD phases.

Data on GCase activity in iPD subjects are controversial. Indeed, a study on a Dutch cohort of *de novo* iPD patients (van Dijk et al., 2013) reported only a trend toward a decrease in GCase activity. These mixed results may be attributed to different collection and storage procedures for CSF, but also to the intrinsic instability of GCase in CSF (Persichetti et al., 2014).

GCase enzymatic activity is also detectable in leucocytes isolated from peripheral blood, which can be obtained with a far simpler and less invasive procedure than CSF. Raghavan et al. (1980) first developed a reliable and reproducible assay technique to measure GCase enzymatic activity in human leukocytes, by using the artificial substrate 4MU-/3-glucoside. Kim et al. (2016) used this technique to explore if GCase activity was altered in the leucocytes of iPD and subjects with PD associated to a genetic mutations. They did not find differences in GCase activity between groups of patients, but GCase activity was positively associated with disease duration in iPD. A reduction of GCase activity has also been observed in blood samples from both iPD and GBA-PD patients (Alcalay et al., 2015). In these cohorts, the authors tested the association between GCase enzymatic activity and PD disease severity. They reported that in iPD patients, higher GCase enzymatic activity was correlated with a longer disease duration and a milder disease progression.

Recently, in a pilot study, Ortega et al. (2016) measured GCase activity from leucocytes isolated from drawn heparinized blood in GBA1 heterozygous and homozygous mutation carriers, with and without PD, and iPD. They observed that GCase activity was significantly lower in GBA-PD than iPD patients and even lower in GD-PD patients, suggesting that GCase activity could be a possible marker of the GBA1 mutated condition.

Other metabolites linked to GCase deficiency have been analyzed in GBA-PD patients. Indeed, GCase deficiency in the brain of PD subjects may also be correlated to pathological alterations in the lipid metabolism (Schapira et al., 2016). In a gas chromatography analysis, free fatty acids in the CSF of GBA-PD patients showed a different pattern compared to iPD and control subjects (Schmid et al., 2012). Alterations of fatty acid metabolites have been found also in the plasma of iPD patients, with an increase of the glucosylcerebroside substrate and different ceramide species (Mielke et al., 2013). These results were confirmed by Pchelina et al. (2018) in a recent study where GBA-PD patients showed increased hexosylsphingosine levels in plasma when compared with iPD and controls.

Alterations of other lysosomal enzymes have been described in iPD subjects. Huebecker et al. (2019) reported that reduction in GCase activity in human post-mortem substantia nigra of iPD were correlated with alterations in lysosomal sphingolipid hydrolases and concomitant glycosphingolipid substrates accumulation. In addition, in this study iPD showed a distinctly significant reduction in levels of complex gangliosides (e.g., GM1a) in substantia nigra, as well as in CSF and serum. Same findings, albeit less marked, were also observed in elderly controls as well as in subjects with RBD (Huebecker et al., 2019).

Although cognitive deficits are frequently reported in GBA-PD, CSF levels of tau and β-amyloid—which are altered in patients with Alzheimer’s disease—were unchanged in GBA-PD patients (Beavan and Schapira, 2013). However, higher baseline CSF levels of p-Tau in GBA-PD patients seem to be related with increased deterioration of cognitive functions over time (24 months; Brockmann et al., 2015a). Moreover, by assessing CSF profiles longitudinally, GBA-PD showed lower levels of Aβ1–42, t-Tau, and p-Tau than control subjects and LRRK2-PD.

Finally, alpha-synuclein was also measured in plasma and in CSF in subjects carrying GBA1 mutations. Parnetti et al. (2014) evaluated alpha-synuclein levels in CSF of iPD subjects, showing decreased levels of total alpha-synuclein in iPD compared with healthy controls in contrast to an increased level of oligomeric form of alpha-synuclein and a higher oligomeric/total alpha-syn ratio in iPD than controls. In the same study they also revealed that in PD patients carrying GBA1 mutations, CSF levels of total and oligomeric alpha-synuclein were higher than iPD (Parnetti et al., 2014).

Moreover, by combining GCase activity, oligomeric/total alpha-synuclein ratio in CSF, and age they discriminated PD patients from controls with a high sensitivity (82%) and specificity (71%; Parnetti et al., 2014).

Nuzhnyi et al. (2015) showed a negative association between plasma oligomeric alpha-synuclein and GCase activity in leucocytes from GD patients, speculating that the reduced GCase activity may be correlated to the peripheral aggregation of toxic alpha-synuclein species.

Recently, Lerche et al. (2020) showed reduced CSF levels of total alpha-synuclein in GBA-PD compared to iPD and controls. Moreover, in this study, GBA-PD subjects bearing a severe mutation showed lower CSF levels of total alpha-synuclein than GBA-PD with mild or risk variant mutations or iPD patients.

Overall, reduced CSF alpha-synuclein levels might reflect its accumulation in the brain, but further studies are warranted to better understand the meaning of alpha-synuclein changes in the CSF of PD patients carrying different pathogenic GBA1 mutations.

## Therapeutic Strategy for GBA-PD Patients

GBA-PD individuals show, especially in the initial phases of the disease, a good response to L-Dopa, although motor symptoms seem to progress more rapidly than in iPD. Therefore, a specific therapeutic management should be considered in these patients.

A recent article investigating the outcomes of treatment with deep brain stimulation in a cohort of GBA-PD patients after a 7.5-year follow-up demonstrated similar outcomes compared to iPD in terms of motor symptoms, whereas cognitive deterioration and non-motor features were more represented among GBA-PD patients (Lythe et al., 2017). However, because of the positive effects on motor impairment, deep brain stimulation should also be considered as a suitable therapeutic option for these patients.

Despite the availability of successful treatments for systemic manifestations in GD patients, these approaches do not affect glycosphingolipid accumulation in the central nervous system, because they are not able to cross the blood-brain barrier. Current experimental approaches available in clinical trials (Table 1) are founded on the main hypothesis that the loss of function determined by GBA1 mutations causes abnormal glycosphingolipid accumulation, leading to neuronal dysfunction and associated proteinopathy. Increasing GCase activity *via* small chaperones showed good efficacy in modulating pathological alterations in models of disease. In order to address this pathogenetic mechanism, two different chaperones that increase GCase activity in the brain were investigated. The mucolytic ambroxol was described to improve GCase enzymatic activity and reduce total and phosphorylated alpha-synuclein levels in models of PD (McNeill et al., 2014; Ambrosi et al., 2015). Currently, two clinical trials assessing the safety, tolerability and efficacy of ambroxol are ongoing. In 2016, a phase II, single-center (Canada), randomized placebo-controlled, double-blind trial started to assess the safety, tolerability, and efficacy of ambroxol on cognitive and motor symptoms of PD Dementia (PDD; ClinicalTrials.gov NCT02914366). Preliminary results showed that higher dose ambroxol are effective and safe in PDD (Silveira et al., 2019). A similar approach is being tested by Schapira’s group in a cohort of GBA-PD patients (ClinicalTrials.gov: NCT02941822).

**Table 1:** Disease-modifying therapies in active GBA-PD trials.

Targeting mechanism	Mechanism of action	Drug	Target population (n. of cases)	Status	Primary outcomes	Enrolling countries	ClinicalTrials. gov/Netherlands Trial Register ID	Sponsor
Increase in GCase activity	GCase activation	Ambroxol	GBA-PD (n.10) PD (n.10)	Phase II, Not placebo controlled	Safety, Tolerability PK/PD	UK	NCT02941822	UCL and Cure Parkinson’s Trust
Increase in GCase activity	GCase activation	Ambroxol	PD-D (n.75)	Phase II, Placebo controlled	Safety, Tolerability PK/PD	Canada	NCT02914366	Lawson Health Research Institute and Weston Foundation
Increase in GCase activity	GCase activation	LTI-291	GBA-PD (n.15) PD (n.15)	Phase I, Placebo controlled	Safety, Tolerability PK/PD	Netherlands	NL7061	LTI, Allergan
Reduction of GCase-related glycosphingolipids	Glucosylcer-amide synthase inhibitor	Venglustat	GBA-PD (n.270)	Phase II, Placebo controlled	Change in MDS-UPDRS parts II & III	Austria Canada, France Germany Israel Italy, Japan Norway Portugal Singapore Spain Sweden Taiwan UK USA	NCT02906020	Sanofi-Genzyme

*PD, Parkinson Disease; PDD, PD dementia; GBA-PD, GBA1-associated PD; PK/PD, Pharmacokinetics, and Pharmacodynamics; MDS-UPDRS, Movement Disorder Society-Unified Parkinson’s Disease Rating Scale*.

Another approach is being tested in the Netherlands by Allergan in a phase I clinical trial with LTI-291, a non-inhibitory chaperone of GCase able to both increase its catalytic activity and extend its half-life (Nederlands Trial Register: NL7061).

An alternative therapeutic strategy that is being explored is to reduce the accumulation of glucosylceramide (the substrate degraded by the normal GCase) by targeting the enzyme glucosylceramide synthetase. The substrate reduction restored glycosphingolipid balance and reduced alpha-synuclein accumulation in neuronal cells of PD with and without GBA1 mutations (Kim et al., 2016; Zunke et al., 2018). In mouse models of GBA-related synucleinopathy, the use of a glucosylceramide synthetase inhibitor improved alpha-synuclein degradation and the behavioral alterations (Sardi et al., 2017). Based on these findings, a multicenter phase II, randomized, double-blind, placebo-controlled study supported by Sanofi-Genzyme, is investigating the safety, pharmacokinetics, and pharmacodynamics of Venglustat (GZ/SAR402671), an oral inhibitor of glucosylceramide synthase in GBA1 carriers with early-stage PD (MOVES-PD study: NCT02906020).

Gene therapy approaches are also being developed as new therapeutic strategies for PD. The most common mechanism used to cross the blood-brain barrier is the delivery of genes *via* viral vectors to the affected areas of the brain. The most common vector used in preclinical studies on animal models in relation to GBA1 is the adeno-associated virus (AAV). Based on the assumption that a decrease in GCase activity might favor alpha-synuclein accumulation and synucleinopathy development, AAV- mediated GBA1 delivery has been tested in mouse models of GD-related PD to assess its effects on GCase and alpha-synuclein modulation (Sardi et al., 2011, 2013; Rocha et al., 2015b). Sardi et al. (2011) first demonstrated that treatment with AAV-GBA1 reduced alpha-synuclein levels, increased GCase activity, reduced glucosylsphingosine and glucoceramide levels, and improved the cognitive deficits in a pre-symptomatic mouse model of GD. The same author replicated the experiment in a symptomatic mouse model of GD-associated PD (Sardi et al., 2013). In this case, the increased GCase was able to modulate the aberrant lipids accumulations, reduce alpha-synuclein and tau aggregation, and ameliorate cognitive functions. When tested in an A53T alpha-synuclein mouse model, the increase in GCase levels reduced alpha-synuclein levels, demonstrating the relationship between alpha-synuclein and GCase activity and suggesting that this approach may also be beneficial for PD without GBA1 mutations (Sardi et al., 2013).

Rocha et al. (2015b) also demonstrated the neuroprotective effects of GBA1 gene transfer in rodent models of PD. They observed that in a transgenic mouse model overexpressing wild-type alpha-synuclein, the increased GCase activity was able to reduce alpha-synuclein accumulation in the substantia nigra and striatum by improving insoluble alpha-synuclein clearance. Moreover, in a rat model of selective dopamine neuron degeneration, they showed that the overexpression of GBA1 preserved dopaminergic neurons from neurodegeneration (Rocha et al., 2015b).

Recently, Morabito et al. (2017) developed a non-invasive approach for the AAV delivery, using intravenous injection. This method has the advantage to treat non-selective brain areas and prolong gene overexpression. Transduction of A53T- SNCA transgenic mice with AAV-PHP.B-GBA1 restored GCase activity and improved alpha-synuclein pathology (Morabito et al., 2017).

Interestingly, Massaro et al. (2019) tested a gene therapy approach on a neuronopathic Gaucher disease mouse model. Authors demonstrated that fetal intracranial injections of AAV-GBA1 increased GCase activity, reduced neuroinflammation, blocked neurodegeneration, and improved motor and systemic symptoms (Massaro et al., 2019). Finally, the same authors assessed the effects of a systemic AAV-GBA1 injection, showing prolongation of the life span of treated mice, reduction of visceral and motor symptoms, and protection from fatal neurodegeneration (Massaro et al., 2020).

Gene therapy may therefore hold fascinating promises for PD patients with and without GBA1 mutations, but clinical trials to assess this type of approach will be needed.

## Counseling and Genetic Testing Strategy in GBA1 Positive Individuals

Although genetic information could potentially help in the diagnosis, prognosis, and treatment of PD, clinical practice guidelines for PD genetic testing are not available yet, and genetic analyses are still limited in PD patients. In the past, the majority of the medical community was reluctant to recommend genetic testing for PD, which was mostly restricted to familial PD. However, in the last few years, the recent development of clinical trials targeting patients with GBA-PD and the launch of personalized medicine has prompted interest in receiving genotype information and increased the demand for genetic counselling regarding GBA1 mutations. In this view, the European Federation of Neurological Societies re-evaluated the recommendations for genetic testing, proposing that it should be considered for PD firstly when the following risk factors are present:

(1) Family history of PD; (2) early onset of PD (before 50 years old); or (3) individual among “population at risk,” such as AJ individuals.

Genetic screening should be also performed to: (1) stratify patients into subtypes to better understand the disease process; and (2) provide patients with the opportunity to participate in clinical trials with experimental therapies designed for patients with specific genotypes (Payne et al., 2019). For all these reasons, genetic counselling should be warranted and implemented.

## Conclusion

GBA1 mutations play a pivotal role in the molecular pathogenesis of PD and are considered one of the most frequent risk factors for the disease. Increasing evidence suggests that GCase deficiency might also have a role in iPD.

The pathobiological mechanisms underlying the link between a defective function of GCase and the development of PD are still poorly understood, but interactions with alpha-synuclein, lysosomal dysfunction, ER stress, and neuroinflammation may all play a crucial role.

Further studies on large GBA1-positive populations will be needed to precisely identify the biological factors that link GBA1 mutations to PD. This will improve the diagnostic and caring process, possibly leading to the discovery of new therapeutic targets to engage in the frame of an innovative, personalized approach to PD therapy.

## Author Contributions

All authors listed have made a substantial, direct and intellectual contribution to the work, and approved it for publication.

## Conflict of Interest

The authors declare that the research was conducted in the absence of any commercial or financial relationships that could be construed as a potential conflict of interest.
